# CD146 Expression Correlates with Epithelial-Mesenchymal Transition Markers and a Poor Prognosis in Gastric Cancer

**DOI:** 10.3390/ijms13056399

**Published:** 2012-05-23

**Authors:** Wen-Fang Liu, Shu-Rong Ji, Jian-Jun Sun, Yi Zhang, Zhong-Yan Liu, Ai-Bin Liang, Hua-Zong Zeng

**Affiliations:** 1 Department of General Surgery, Tongji Hospital of Tongji University, Shanghai 200065, China; E-Mail: Wenfangliu22@163.com; 2 Central Laboratory, Tongji Hospital of Tongji University, Shanghai 200065, China; E-Mails: shurongji@126.com (S.-R.J.); Jianjunsun12@yeah.net (J.-J.S.); yi_zhanglab@163.com (Y.Z.); zhyanliu@163.com (Z.-Y.L.); 3 School of Life Sciences and Technology, Tongji University, Shanghai 200092, China

**Keywords:** CD146, gastric cancer, epithelial-mesenchymal transition, prognosis, immunohistochemistry

## Abstract

CD146 has been regarded as a novel potential therapeutic target for multiple cancers. The aim of the study was to investigate the expression of CD146 in gastric cancer and evaluate its clinical-pathological and prognostic significance. The expression of CD146 and three epithelial-mesenchymal transition (EMT)-related proteins (E-cadherin, β-catenin and vimentin) was examined in 144 gastric cancers by immunohistochemistry. Fifty-nine cases (41.0%) were defined as positive for CD146 expression. We found that CD146 expression correlated positively with lymph node involvement and a poor prognosis, and retained an independent prognostic factor for gastric cancer patients. Furthermore, positive expression of CD146 was strongly associated with loss of the epithelial marker E-cadherin and acquisition of the expression of the mesenchymal markers nuclear β-catenin and vimentin. These findings suggest that CD146 might promote EMT and progression in gastric cancer, and thus may be a potential therapeutic target for patients with gastric cancers.

## 1. Introduction

Gastric cancer is one of the most frequent cancers worldwide. In China, gastric cancer ranks the third most common cancer [1]. Although the establishment of screening, early diagnosis and curative operation has increased survivals significantly, recurrence and metastasis remains a great challenge for patients with gastric cancer. In this context, there is an urgent need to find novel cancer-related factors to be used as biomarkers for diagnosis and therapeutic targets of gastric cancer.

CD146, also known as MCAM, Mel-CAM, MUC18, S-endo1, was first identified as a cell-adhesion molecule specific for melanoma [2]. CD146 plays a critical pro-migratory role in the vascular system, normal development, and tumor progression patterning [3,4]. Overexpression of CD146 has been discovered in many cancers, including melanoma, prostate cancer, epithelial ovarian cancer, and breast cancer, and is associated with tumor progression [5–7]. The many underlying mechanisms of CD146 involved in cancer progression are now being elucidated. The epithelial to mesenchymal transition (EMT) is a mechanism by which tumor cells acquire characteristics which could increase their metastatic potential, and targeting EMT processes is therefore a promising strategy to block the transition to metastatic phenotype and improve the patients' outcome. A panel of EMT markers has been identified including up-regulation of mesenchymal vimentin, N-cadherin, and nuclear β-catenin, and down-regulation of epithelial markers E-cadherin. Most recently, Zeng *et al.* showed a striking association between CD146 and EMT in breast cancer [8]. Firstly, CD146 could induce EMT of the breast cancer cells via the activation of RhoA and up-regulation of the key EMT transcriptional factor Slug. Secondly, overexpression of CD146 was strongly associated with E-cadherin down-regulation in triple-negative breast cancers (TNBC) samples. Thus, CD146 could be an attractive target for cancer therapy and a potential biomarker for cancer prognosis.

However, currently, no study has reported CD146 expression in gastric cancer patients. Therefore, in the present study, the expression of CD146 in gastric cancer and its association with clinicopathological parameters and prognosis were evaluated. Furthermore, the association of CD146 and three EMT markers (E-cadherin, β-catenin and vimentin) was also investigated.

## 2. Results and Discussion

### 2.1. Expression of CD146 and EMT Markers in Gastric Cancer

**Figure 1:**
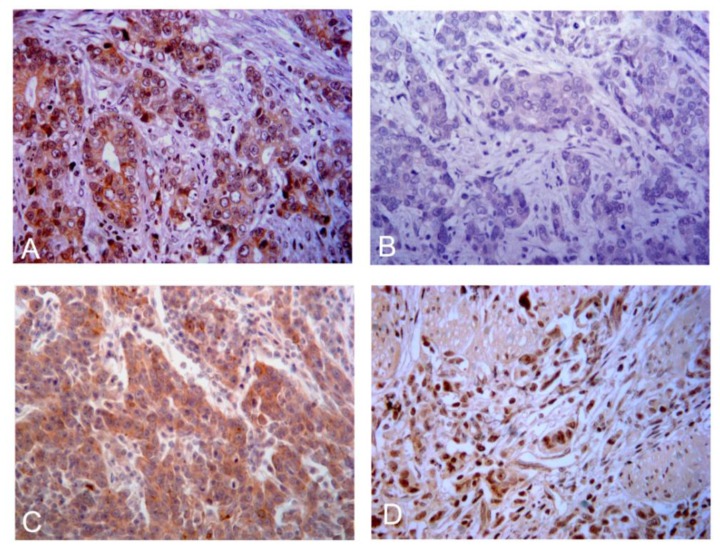
Representative images showing cytoplasmic and membranous CD146 expression (**A**); E-cadherin loss (**B**); cytoplasmic vimentin expression (**C**); and nuclear β-catenin expression (**D**) in gastric cancers (×200 magnitude).

Immunohistochemistry revealed that CD146 positive staining was localized in the membrane and cytoplasm of tumor cells in gastric cancer. According to the criteria established for immunostaining, 41.0% (59/144) of tumors were positive for CD146 staining. To investigate whether the abnormally high expression of CD146 in gastric cancers accounts for its EMT-like features, we further analyzed correlations between CD146 and three EMT markers. In gastric cancers, epithelial protein loss frequency was 31.2% (45/144) for E-cadherin, and aberrant mesenchymal protein expression frequencies were 56.3% (81/144) for nuclear β-catenin and 56.3% (81/144) for vimentin. Figure 1 shows the representative immunohistochemistry results.

### 2.2. Correlation between CD146 Expression with Clinicopathological Characteristics and EMT Markers in Gastric Cancer

The correlation between CD146 expression status and the clinicopathological characteristics in gastric cancer was further analyzed and the results are summarized in Table 1. Over-expression of CD146 was closely related to lymph node metastasis (*P* < 0.001), but no significant correlation was present with patients’ age, gender, the depth of invasion, pTNM stage and histology. Moreover, the positive expression of CD146 was negatively correlated with E-cadherin (*r* = −0.185, *P* = 0.032) and was positively associated with nuclear β-catenin (*r* = 0.41, *P* < 0.001) and vimentin (*r* = 0.287, *P* = 0.001) expression (Table 2).

### 2.3. Correlation between CD146 and EMT Markers Expression with Gastric Cancer Survival

**Figure 2:**
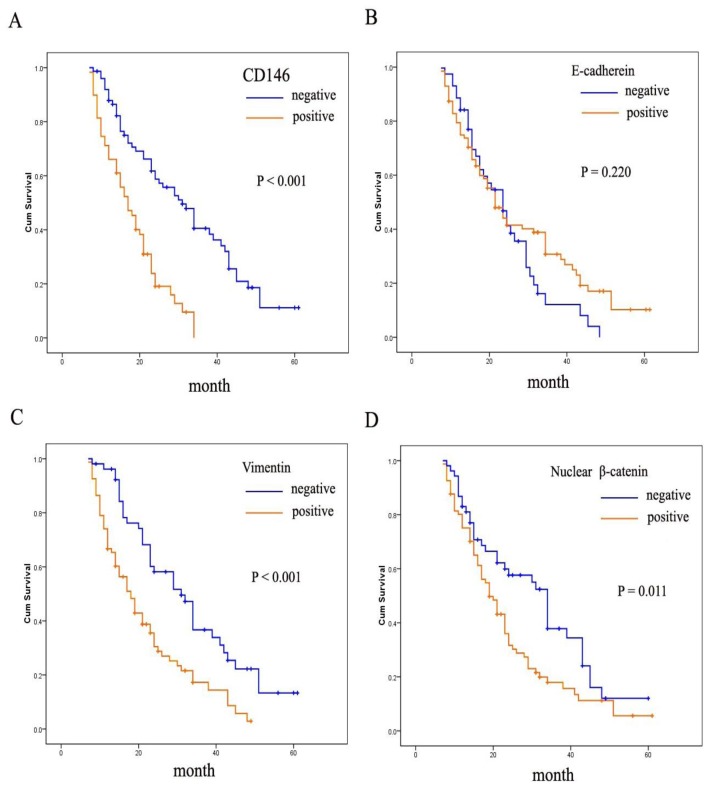
Overall survival curves of patients with gastric cancer according to the immunostaining results of CD146 (**A**); loss of E-cadherin (**B**); aberrant expression of vimentin (**C**); and β-catenin (**D**); determined by the Kaplan–Meier analysis.

Patients with positive CD146 expression showed a more unfavorable prognosis than those with no expression (Figure 2A). This was also true for increased expression of vimentin and nuclear β-catenin (Figure 2C,D). However the absence of E-cadherin was associated with no significant difference in outcomes (Figure 2B). Moreover, as seen in Table 3, multivariate Cox analysis showed that CD146 over-expression was an independent prognostic factor for patients with gastric cancer (*P* = 0.027).

### 2.4. Discussion

CD146, a cell-cell or cell-matrix adhesion molecule, was first described in melanomas. Although, a few studies have indicated its role as a tumor suppressor [9], accumulating evidence supports that CD146 acts as a pro-metastatic factor [10]. In particular, many recent studies have identified that the high expression of CD146 has been associated with metastatic progression in prostate cancer, breast cancer and ovarian cancer. A critical question raised was whether CD146 expression clinically correlated with gastric cancer progression. To address this issue, we performed immunohistochemistry to detect CD146 expression in 144 human primary gastric cancers.

In this study, our data demonstrated that a subset (41.0%) of human primary gastric cancers expressed CD146 proteins in the epithelial compartment. CD146 expression correlated with lymph node metastasis status, and was associated with a poor overall survival. Cox model analysis revealed that CD146 was an independent prognostic factor for gastric patients. Our findings indicated that similar to other solid cancers recently investigated, CD146 was also associated with tumor metastasis and poor prognosis in gastric cancer, and further enhanced the role of CD146 in the progression of cancers.

*In vitro* assays revealed that CD146 silencing in cancer cells resulted in down-regulation of mesenchymal markers (vimentin and fibronectin) and up-regulation of the epithelial marker (E-cadherin) [7,8]. Consistent with such changes in EMT markers, functional assays revealed that CD146 silencing was also accompanied by decreased migration and invasion abilities of cancer cells. Zeng *et al.* [8] suggests that RhoA pathway-mediated EMT transcriptional factor Slug activation could be a key mechanism for the EMT induced by CD146 in cancer cells. Moreover, Zeng *et al.* [8] demonstrated the correlation between CD146 expression and E-cadherin down-regulation in breast cancers. Established drivers of EMT include activated transforming growth factor b (aTGFb), Snail or Zeb1 [11]. In this study, using three EMT markers, we further confirmed that CD146 over-expression was associated not only with E-cadherin down-regulation, but also with acquisition of the expression of the mesenchymal markers nuclear β-catenin and vimentin. Our data further indicated that CD146 was strongly associated with EMT protein changes in clinical cancer samples.

Previous studies have confirmed that EMT is associated with the progressive phenotypes and poor outcome in gastric cancer [12,13]. Considering that CD146 is closely related to EMT and progression of gastric cancer, we suggest that CD146 inhibition might offer a promising target for EMT reversion and prevention of metastatic progression and invasion in gastric cancer.

## 3. Experimental Section

### 3.1. Patients

A total of 144 patients with histologically confirmed gastric adenocarcinoma invading the submucosal layer or deeper were retrospectively included in this study. All patients received the curative gastrectomy with lymph node dissection at Tongji Hospital of Tongji University, Shanghai, China between January 2006 and September 2009. All the patients received postoperative chemotherapy regimen based on 5-fluorouracil plus leucovorin. No patient received preoperative chemotherapy and/or radiotherapy. The clinicopathological characteristics of the patients, including age, gender, pathological TNM stage (pTNM stage), histology, depth, and lymph node metastasis status, are seen in Table 1. Follow-up information, including patient outcome and the time interval between the date of surgical resection and the date of the cancer-related death, was collected. Those cases lost to follow-up and deaths from causes other than gastric cancer were regarded as censored data for the analysis of survival. The study was approved by our local ethics committees. Specimens were obtained with informed consent.

### 3.2. Immunohistochemistry

Immunohistochemistry was performed to detect the expression of CD146, E-cadherin, β-catenin and vimentin in formalin-fixed paraffin-embedded gastric cancer specimens. Briefly, 4 μm thick sections were deparaffinized with xylene, then were incubated with 0.3% hydrogen peroxide for 30 min to block the endogenous peroxidases. After treatment with normal goat serum for 1 h to block nonspecific binding, primary antibodies against CD146 (Abcam, 1:100, Cat. # ab75769), E-cadherin (Santa Cruz, 1:200, Cat. # sc-8426), β-catenin (Santa Cruz, 1:100, Cat. # sc-7199) and vimentin (Santz Cruz, 1:100, Cat. # sc-32322) were added, respectively. Primary antibodies were detected using a HRP Envision System kit (Dako, Gene Co. Ltd., Shanghai, China), and then were counterstained with hematoxylin. For the negative controls, the primary antibodies were replaced by mouse or rabbit IgG (Santa Cruz).

The results of immunostaining were evaluated based upon the percentage of mild or strong staining cancer cells to the total cancer cells. According to the criteria established in the previous studies, immunohistochemistry results were considered to be positive when the percentage of mild staining cells was greater than >10% of tumor cells. β-catenin nuclear localization was considered aberrant expression.

### 3.3. Statistical Analysis

The relationship between CD146 expression and clinicopathologic variables was evaluated using Chi-square test. The nonparametric Spearman rank correlation coefficient was applied to analyze the relationship between CD146 and EMT markers. Overall survival of patients was estimated by the Kaplan-Meier method, and the statistical significance of the differences were compared by the log-rank test. A Cox proportional hazards regression model was used for the multivariate analysis. All the statistical analyses were performed with SPSS 16.0 (SPSS: Chicago, IL, USA, 2008). *P* ≤ 0.05 was considered statistically significant.

## 4. Conclusions

In conclusion, we found that CD146 was associated with lymph node metastasis and EMT marker changes in gastric cancer, and was also a novel prognostic factor. The findings are consistent with the expression of CD146 in other cancers such as breast cancer. We suggest that CD146 could be also used as a potential therapeutic target for patients with gastric cancers.

## Figures and Tables

**Table 1:** Correlations between CD146 with clinicopathologic characteristics.

		CD146		
		
Clinical Factors	*n*	Negative (*n* = 75)	Positive (*n* = 59)	*P* Value
**Age (year)**				
<65	96	54	42	0.917
≥65	38	21	17	
**Gender**				
Male	90	52	38	0.547
Female	44	23	21	
**Depth**				
T1/T2	27	16	11	0.700
T3/T4	107	59	48	
**Nodal involvement**				
Yes	93	42	51	<0.001
No	41	33	8	
**pTNM stage**				
1/2	44	23	21	0.547
3/4	90	52	38	
**Histology**				
Differentiated	75	46	29	0.159
Undifferentiated	59	29	30	

**Table 2:** Correlations between CD146 with EMT markers.

EMT Markers	*n*	CD146			

		Negative	Positive	*r*	*P* Value
**Ecadherin**					
Negative	89	44	45	−0.185	0.032
Positive	45	31	14		
**Nuclear β-catenin**					
Negative	53	43	10	0.41	<0.001
Positive	81	32	49		
**Vimentin**					
Negative	53	39	14	0.287	0.001
Positive	81	36	45		

**Table 3:** Multivariate analysis with regard to overall survival.

Parameter	Multivariate Analysis	

	*p*-Value	*RR* (95% CI)
**Age** (<65 *vs.* ≥65)	0.332	1.257 (0.792–1.996)
**Gender** (male *vs.* female)	0.579	1.137 (0.722–1.792)
**Depth** (T1/T2 *vs.* T3/T4)	0.105	0.560 (0.278–1.128)
**Lymph node metastasis** (yes *vs.* no)	0.001	3.256 (1.595–6.647)
**Histology** (differentiated *vs.* undifferentiated)	0.862	1.038 (0.685–1.572)
**pTNM stage** (3/4 *vs.* 1/2)	0.141	1.442 (0.886–2.344)
**E-cadherin** (Positive *vs.* negative)	0.715	1.086 (0.697–1.693)
**Nuclear β-catenin** (positive *vs.* negative)	0.002	2.096 (1.312–3.347)
**Vimentin** (positive *vs.* negative)	0.037	1.664 (1.031–2.686)
**CD146** (positive *vs.* negative)	0.027	1.744 (1.066–2.853)
